# '*I should not feed such a weak woman*'. Intimate partner violence among women living with podoconiosis: A qualitative study in northern Ethiopia

**DOI:** 10.1371/journal.pone.0207571

**Published:** 2018-12-06

**Authors:** Girmay Tsegay, Kebede Deribe, Negussie Deyessa, Adamu Addissie, Gail Davey, Max Cooper, Mei L. Trueba

**Affiliations:** 1 College of Medicine and Health Sciences, Debre Markos University, Debre Markos, Ethiopia; 2 Wellcome Trust Brighton & Sussex Centre for Global Health Research, Brighton & Sussex Medical School, Falmer, Brighton, United Kingdom; 3 School of Public Health, Addis Ababa University, Addis Ababa, Ethiopia; USC Keck School of Medicine, Institute for Global Health, UNITED STATES

## Abstract

**Background:**

Intimate Partner Violence (IPV) is a serious, preventable public health problem that affects millions of people worldwide. Research indicates that adults suffering from long term, disabling conditions are more likely to be victims of IPV due to the intersection of disease-associated stigma and discrimination. IPV in turn is known to worsen the overall health and wellbeing of those affected by it. Little research however explores the relationship between neglected tropical diseases such as podoconiosis and IPV. This study explores the relationship between IPV and podoconiosis in northern Ethiopia with the aim of identifying new avenues for limiting disability and promoting the wellbeing of people affected by this neglected tropical disease.

**Methods:**

The study was conducted in East and West Gojjam zones, located in the Amhara Regional State of Ethiopia. Research participants were first screened using the domestic violence screening tool Hurt-Insult-Threaten-Scream (HITS). Data were collected by native speakers of the local language (Amharic) in the form of semi-structured interviews during January and February 2016. Thematic and content data analysis was carried out, using the Open Code 3.4 qualitative data analysis software for coding.

**Results:**

A total of 15 women living with podoconiosis and experiencing IPV were interviewed (aged 31 to 75). Women experienced different forms of IPV, including beatings (with or without an object), insults, name calling, undermining, denial of equal rights over common assets, movement monitoring, cheating, abandonment, forced divorce, obstruction of health care access, inhibition of decision-making and sexual coercion. Podoconiosis increases the frequency and severity of IPV and in occasions shapes a change from physical to psychological and financial violence. In turn, frequent episodes of IPV worsen disease outcomes and contribute to disease persistence in the region, in that these impede women’s ability to manage the disease and help perpetuate the conditions of poverty that influence disease onset.

**Conclusions:**

Women living with podoconiosis are victims of various, overlapping forms of IPV that negatively impact their health and wellbeing. Poverty, scarce IPV prevention services in the area together with a social acceptance of IPV and these women’s decreased ability to work due to the debilitating effects of podoconiosis and childcare responsibilities frequently prompt these women to tolerate IPV and remain in abusive relationships. Tackling disease-associated taboo and stigma, developing accessible IPV interventions, working towards greater gender equality at the household and societal levels and developing sustainable strategies for improving the socio-economic assets of women affected by podoconiosis are all necessary to both prevent IPV and to improve disease outcome.

## Background

Intimate Partner Violence (IPV), defined as physical, sexual, or psychological harm by a current or former partner or spouse [1], is a serious but preventable public health problem that affects millions of people worldwide. It is one of the most common forms of violence against women [1], and can vary in frequency and severity [2]. Current research recognizes various types of intimate partner violence, including physical, psychological, emotional, sexual, financial and threats of violence [3–5] In Ethiopia alone, the World Health Organization (WHO) estimates that 71% of women suffer physical and/or sexual violence by an intimate partner in their lifetime [6].

It is now accepted that adults suffering from long term conditions or disabilities are 1.5 times more likely to be victims of violence than those without a disability [7]. According to the abundant body of literature on IPV among women living with HIV, this is due to the intersection of disease-associated stigma and discrimination [8–10]. This is of special importance in the case of podoconiosis, given that people with podoconiosis frequently experience stigma in their day to day interactions with family members [11, 12]. Podoconiosis (endemic non-filarial elephantiasis) is a neglected tropical disease (NTD) that can affect individuals exposed to red-clay soil derived from volcanic rock. Equally affecting men and women this disease causes severe swelling of the lower legs and disability [13]. Consequently, people are both stigmatized and discriminated against. This stigma is associated with decreased ability to work, and also with misconceptions surrounding the causes of the disease–often interpreted locally as a punishment from God [11, 14].

In endemic areas, podoconiosis is one of the most common causes of stigmatization [12, 15]. People living with podoconiosis frequently experience stigma in their day-to-day interactions with family members, and the disease ultimately leads to social exclusion of the affected individuals and their families. In studies related to stigmatization, people living with podoconiosis commonly reported that they had considered suicide in response to discrimination and prejudice, particularly in interpersonal interactions [16, 17]. Forced divorce, insults and exclusion at and from social events were some of the most commonly mentioned forms of enacted stigma reported by informants. The most pronounced manifestations of social stigma in endemic areas are being unable to marry, and being excluded from school, church, and social events [16, 17]. Prejudice and discrimination by family members and deprivation of emotional and material support are common in the region [16, 17].

The most recent global estimates for IPV reveal that 30% of women aged 15 and over have experienced physical and/or sexual IPV in their lifetime [18]. Studies in Ethiopia have also shown that between one-half to two-thirds of women experience one or more forms of spousal abuse at least once in their lifetime [19, 20]. However, even though a significant association between lifetime experience of IPV and self-reported poor health and specific health problems is recognized [21], little research explores the relationship between IPV and neglected long term conditions such as podoconiosis. Yet, such an exploration is important both for improving podoconiosis outcomes and for diminishing IPV prevalence in the region. With this purpose this study aimed to explore how podoconiosis influences IPV and how, in turn, IPV influences podoconiosis outcomes. The study also sought to explore the determinants of these relationships in order to establish locally relevant avenues for action.

## Methods

### Study area

The study was conducted in East and West Gojjam zones, in the Amhara Region of Ethiopia. This region is located in the northwestern part of the country and its land area is estimated to be 170,000 square kilometers with a population density of 110/km^2^. Predominantly rural, the region is made up of subsistence farms and grazing fields. The prevalence of podoconiosis in the study area is 3.3% [22]. Women in the region, as in the rest of the country, occupy low status in society. Although they represent half the population and contribute disproportionately to food production, they have not benefitted equally. Basic rights such as access to land, credit and other productive resources are difficult for women to attain. Women simultaneously experience burdens and deprivations such as longer working days due to being the solely responsible for children and household care, lower levels of education and lack of representation in leadership and decision-making positions [23, 24]. Several other poverty-related and socio-cultural factors shape many of the hardships lived by women in this region. These include socially condoned violence against girls and women in the form of female genital mutilation/cutting (FGM/C), early marriage, abduction and eventual rape, as well as forced marriage followed by pregnancy and childbirth in the teenage years [25, 26].

The East Gojjam zone has an estimated population of 2,153,937 people and an area of 14,000 square kilometers, giving a population density of 153.80/km^2^ [27]. The West Gojjam zone has an estimated population of 2,106,596 people, an area of 13,300 square kilometers and a population density of 158.25/km^2^ [27]. The study was conducted through purposively-selected IOCC (International Orthodox Christian Charities) treatment sites. IOCC is the only podoconiosis treatment and prevention service provider in the region, and has several sites in these two zones.

### Study design and sampling

We conducted an exploratory qualitative study using semi-structured interviews among women with podoconiosis and a history of IPV. We used purposive sampling to select study participants. The study included all female podoconiosis patients who visited the IOCC treatments centers, were older than 18 and scored greater than 10 using the HITS tool. We used this screening method to identify women who had experienced IPV during their lifetime. The HITS tool screens for physical harm, insults, threats and verbal abuse. Each item is scored from 1–5 [28, 29], and scores range from 4–20. In accordance with Sherin *et al*’s findings [29], a score greater than 10 was considered to accurately represent existence of IPV and thus determined eligibility for the semi-structured interview. A total of 105 women were screened for IPV by an experienced female sociologist (EL) from Debre Markos University, of which 84 were eligible for interview and 15 consented to the interviews.

### Data collection

Data were collected during January and February 2016 by experienced female data collectors who were native speakers of the local language, Amharic. Women attending for treatment were contacted after their treatment was completed, and interviews lasting approximately 45 minutes were held in a separate place. During interviews women were asked about their experiences of IPV and their coping and response mechanisms. An experienced note-taker was hired to take notes during interviews, and audio recording was also used with prior consent of the informants. A unique identifying number was given to all informants in advance to link the data obtained through interviews.

### Data analysis

Audiotape recordings and notes were first transcribed into Amharic and then translated into English before conducting the thematic and content analysis of data. We used Open Code qualitative software program to assist with analysis. The software allowed us to assign codes to raw data and to assign strict defining parameters to the codes, maximizing consistency in the coding process.

We used inductive coding as a guiding analytic framework and therefore coding categories and themes derived directly from the collected data. Specifically, texts were coded according to categories and themes that emerged from the interviews. As a text was assigned, codes were defined using strict parameters; subsequent text vignettes were coded accordingly. Once all of the interviews and notes were coded, we again classified the codes into overarching themes that directly corresponded to the primary research aim. Then within each of these broad themes, data were organized into narrower constructs, concepts, and categories that allowed for data interpretation. To manage issues associated with researcher bias data analysis was conducted independently by three authors (GT, MC, MLT), with disagreements resolved by group discussion after re-evaluation of collected data.

### Ethical considerations

Study participants were recruited once they were fully aware of the purpose of the study and the methods of data collection. They were asked to provide written consent. When this was not possible potential participants were allowed to provide oral consent (witnessed by IOCC staff). Potential participants were clearly informed that they had the right to stop the screening and interview at any time or to skip questions they did not want to answer. It was also explained that declining participation would not affect their access to IOCC treatment services or the quality of the care received. They were also informed that the data collected would be held in a private place, that identifiable data would not be viewed by any third party except the principal investigators, and that only the age of participants (together with pseudonyms) would be used when identifying quotes in the manuscript. All women who had experienced IPV were referred for further counselling at Debre Markos Referral Hospital, to relevant social services and to the local police administration as appropriate. The Ethical Clearance Committee of the College of Medicine and Health Sciences at Debre Markos University approved the study including the method of recording of informed consent.

## Results

Out of the 105 women screened using the HITS tool, a total of 84 scored greater than 10 (80%), 15 of whom consented to the interviews.

Fifteen women were interviewed, aged between 31 and 75 (see Table 1 below). Eleven of them were divorced or separated at the time of interview and were living with their extended families (27%), alone (18%) or on their own with their children (55%). All interviewed women were poor rural dwellers that combined begging (26.7%) or farming and care of livestock with household and childcare work.

**Table 1:** Socio-demographic characteristics of research participants.

Participant code	Pseudonym	Age	Employment	Area of residence	Marital status	Household composition
1	Selam	50	Farmer	Yejubie	Married	Living with husband and 2 children
2	Genet	40	Farmer	Finote-selam	Divorced	Lives with her 3 children
3	Aster	53	Seasonal farmer & beggar	Debre Elias	Divorced	Lives with her 3 children
4	Tena	45	Farmer	Yejubie	Divorced	Lives with her sister and her 2 children
5	Ewnet	41	Farmer	Yejubie	Divorced	Lives with her 3 children
6	Tsega	45	Seasonal farmer & beggar	Bure	Divorced	Lives with her daughter
7	Fikirte	50	Daily laborer & beggar	Bure	Divorced	Lives alone
8	Abebech	59	Farmer	Debre Elias	Divorced	Lives with her son
9	Bizu	31	Daily laborer	Debre Elias	Married	Living with husband
10	Tiruwork	75	Daily laborer, street vendor (water) & Beggar	Amanuel	Divorced	Lives alone
11	Elfinesh	42	Daily laborer	Amanuel	Divorced	Lives with her aunt and her 2 children
12	Dinberwa	33	Daily laborer	Finote-selam	Separated	Lives with her sister
13	Darimyelesh	45	Daily laborer	Finote-selam	Separated	Lives with 3 children
14	Beletech	45	Daily laborer	Yejubie	Married	Living with husband and 2 children
15	Firehiwot	52	Farmer	Finote-selam	Married	Living with husband

Data indicate that all women were victims of overlapping forms of IPV, mostly beatings (with or without an object), insults, name calling, undermining, monitoring of movements, obstruction of access to common assets cheating and sexual coercion. 87% of interviewed women had experienced IPV prior to disease onset and therefore podoconiosis was not the only determinant of IPV. Data however indicate that podoconiosis did influence an increase in the frequency and severity of IPV and on occasion shaped a change from physical to psychological and financial violence:

“*His violence increased as the disease aggravated.. the problem is the disease because it hinders my ability to work hard…*”

(Selam, aged 50s)

“*When the disease aggravated his insults and threats also aggravated.. he beat me less with the stick but slapped me more and refused to bring water or food… when I was a healthier, he had a good behavior too. But when my disease become worse he started to stay ‘go from this house and let me marry another….I must marry a healthier woman!’*”

(Genet, aged 40)

As illustrated in these quotes (see also the quotes below), men’s frustration at being married to an ‘unhealthy’ woman who cannot always fulfill household and work expectations is a key determinant of IPV among women living with podoconiosis. According to the inductive process of data analysis we organized the results in three overarching themes: a) Types of IPV experienced by women living with podoconiosis; b) Coping strategies and responses; c) Impact of IPV on podoconiosis outcomes and on dependents.

### Types of IPV experienced by women

#### Physical violence

All women reported physical violence from their partners, mainly in the form of beatings (with or without objects), slaps, pushes and by neglecting the physical needs of women. Two women also explained that unless they disappeared out of view and kept silent, their partners would even attempt to kill them using knives or axes, and one woman reported being threatened with the use of a gun:

“*He used to beat me using a stick on the softest part of my body. He was careful not to break me, But, I was badly injured one day*”.

(Ewnet, aged 41)

“*If he held a stick, he used to beat me with it; otherwise, he hit me against a wall. He even threatened me with a gun*”.

(Dinberwa, aged 33).

Women’s decreased ability to work due to the debilitating effects of podoconiosis often triggered episodes of physical violence, although physical violence most often occurred after the partner had been drinking alcohol:

“*…when he arrived home, he was always complaining at everything, saying ‘why didn’t you do this and this?’ and I was telling him ‘you know that I can’t do all the activities in the house and outside the house (in the farmland).’ He used to beat me and say ‘if you can’t, go away from the house!’*”

(Aster, aged 53)

“*He used to work and harvest, sell and drink. Then arriving home he says ‘while I am working hard, you only sit at home, and I should not feed such a weak woman’. He used to attack me with whatever he found around him just like a knife or even an axe. If he got drunk, he would use anything*..”

(Genet, aged 40)

#### Psychological violence

Nine respondents (60%) reported having also suffered from psychological violence. Partners and husbands frequently threatened women, who reported being insulted, shouted at and nagged, both in private and in front of relatives, children and friends. Participants’ partners also used offensive nicknames derived from the physical characteristics of podoconiosis–for example “false banana plant root” (*koba egere) or* “you swollen leg”. Other insults, such as “daughter of a leper”, related to other stigmatizing skin conditions that are often confused with podoconiosis, in this case leprosy. In Ethiopia it is common for people to believe that podoconiosis is caused by a punishment of the gods [14]; for instance, due to the affected person not fulfilling certain social or cosmological rules. This is reflected for instance in the following quote:

“*He used to call me ‘swelled leg’ and ‘devilish woman’… because the disease was given to me by God*”

(Tiruwork, aged 75)

In this sense, behind these nicknames it is reflected the fact that the husband blamed his wife for having a disease that severely restricted the women’s ability to carry out their normal duties (farming, feeding livestock, household, shopping, etc.) or, in the terms used by various participants, ‘what the woman should do’. As illustrated in the quotes below, podoconiosis becomes then a source of inspiration for emotional and psychological IPV in that women with podoconiosis were often accused of being sinful by their husbands and became the target of insults that made reference to some sort of loss of humanity (as if these women were a motionless, fruitless and worthless plant):

“*He insults me saying ‘koba egere’ (false banana root). I am no longer considered a human being*”

(Selam, aged 50)

‘*My husband said to me ‘unless you become healthier, you are nothing to me*’

(Abebech, aged 59)

#### Controlling behaviors

60% of interviewed women were victims of controlling behaviors, which included isolating a woman from her family and friends, monitoring her movements, and restricting her access to financial resources, employment, education or medical care. Some of the participants said that their partners prevented them from visiting their friends and family, delayed access to healthcare services and failed to consult them over decision making about resource-sharing, selling of assets or buying items of importance to the family. Quotes illustrating these controlling behaviors included:

“*I feel angry (…) I had no opportunity to meet with my own family at funerals or marriages ceremonies. I was only serving his relatives. One day, when my mother built a house, I wanted to take tella [local alcohol] to congratulate her. However, he beat me and ordered me to unload the tella. He gave the tella to the cattle*.”

(Tiruwork, aged 75)

“*When I begged him to take me to the health center, he always made excuses. Then the disease got worse as a result of the delay*”

(Genet, aged 40)

“*I never went to the police, but I went to the kebele [local government office] administrator to divide our properties. With the help of the administrator, I got my share of the house which I built by selling my cow. However, he [husband] refused to share the money that he had made from selling our ox*.”

(Tiruwork, aged 75)

“*He would not bring me the household goods I needed or tell me the price of the cereal he sold. If I asked to go to the market with him, he used to refuse, and would not let me go*..”

(Ewnet, aged 41)

#### Sexual coercion

In addition to a range of domestic responsibilities expected from women, a further marital expectation lays in the husband’s desire for frequent sexual intercourse. Where this was unmet it led to domestic tension and strain, increasing the frequency of IPV. Sexual violence is commonly expressed as sexual coercion, but in the case of podoconiosis sexual violence is manifested by extra-marital relations and cheating. 47% of the research participants reported that their husbands avoided sexual intercourse with them and instead sought for it elsewhere. Although extra-marital relations are not uncommon in these regions, this tendency also denotes a desire to be with a “healthier” woman (as illustrated as well in the above quotes). Many of the respondents’ partners had another “wife” living at a distance or accessed sex “at the town”. In these situations, the original wives were typically rejected and eventually abandoned (15%), sometimes resulting in exposure to sexually transmitted infection:

“*He seemed faithful at the beginning but cheated and disappeared after one other woman became pregnant*”

(Fikirte, aged 50)

“*He used to share a bed with many women at the town. He had been passing time (day and night) at the town. He was not eating the food that I prepared. He was always disturbing me by his arguments. You know, when he did all these things, your mind can’t be healthy. This is all about my husband that I marry him at the rural area. After that I decided to lead my life going to the town. Again I became a victim of another disease there. There is no good time for a woman*”

(Elfinesh, aged 42)

### Coping strategies and women’s responses to IPV

Data indicate that IPV was socially tolerated and generally considered as “normal”. This appears to arise from a wider social acceptance of this phenomenon in the region, by the stigma associated with podoconiosis and by women’s low social recognition and lack of economic empowerment. Further drivers of IPV tolerance included fear that unhealthy women could not live alone and needed a man to bring in some household income, a perception of IPV as “my fate” (Genet, aged 40; Firehiwot, aged 52), and most women’s fear that reacting against their husband’s violence would bring even more violence (e.g. Tena, aged 45). Women also put up with IPV in order to protect their children: their responsibilities towards their children influenced their acceptance of intimate partner violence. This is illustrated in the following quotes:

“*My children always advise me ‘if you divorce him, he is going to marry another woman and have children with her, so you are going to let them share your property with us.’*”

(Selam, aged 50)

“*I prefer to keep silent because it would be worst if I gave him a response.. I would prefer to leave the house; however, I stay for my children. Beyond this, my togetherness with my husband is not based on love*”

(Beletech, aged 45)

To prevent episodes of IPV, women try to fulfill their partners’ expectations despite their restricted mobility and cope with episodes of IPV, mostly on their own, by simply keeping quiet and “accepting the storm” (Bizu, aged 31), hiding, or by running to relatives’ or neighbors’ homes when things get worse and doing this is possible. In fact, women’s IPV tolerance is largely influenced by the lack of formal sources of local social support for people experiencing it. Most women are simply left to fend for themselves. Consequently, their coping mechanisms and their responses to IPV are heavily influenced by their social circle’s views on podoconiosis and on IPV. Supportive circles become safety nets that help the woman face the situation and escape from it, while unsupportive circles create tolerance. However, some women eventually do seek to escape these situations regardless of the advice received from their social circles and these decisions depend on the women’s assets, personality and the impact of their particular circumstances on their self-esteem.

“*Since I am physically weak, I try to hide myself inside the house: otherwise, I try to go out to get help from other people. If I’ve already been caught by him, I simply shout for help from the neighbor. If it becomes worse, I go away from the house*”

(Firehiwot, aged 52)

“*There was no equality in my marriage; rather I lived under his control. I had no right to insult him while I was being insulted, and I had no equality to speak back when he spoke. Even when my neighbours listened to me giving a response, they were advising me not to do that, and they were advising me to go and hide inside my house. They and the elders advised me to stop complaining. They tell me I shall worry for my children not to separate them from their family, and the husband will stop his behaviour as his age increase. I simply kept quiet. Years after I left him*”

(Darimyelesh, aged 45)

“*I tried to leave him but my mother and father ordered me to come back… also the elders,,, I finally left him and I said to the elder to marry him*”

(Tena, aged 45).

Help-seeking behaviours are often limited by the reduced mobility that is characteristic of people with podoconiosis. In addition, for most interviewed women, leaving the husband would mean falling into poverty due to reduced ability to work and significantly reduced assets.

### Impact of IPV on disease outcomes and on family members

Women living with podoconiosis are victims of various, overlapping forms of IPV that negatively impact their health and wellbeing. Podoconiosis reinforces the frequency and severity of IPV and on occasion shapes a change from physical to psychological and financial violence–including obstruction to accessing assets. In turn, frequent episodes of IPV worsen disease outcomes in that these impede women’s ability to manage the disease. This is because, as illustrated in some of the above quotes, men often obstructed health care access, but also because they refused to provide women with water, ointments and bandages required for self-care. Women’s inability to rest and keep their legs elevated due to their attempts at fulfilling the household, childcare and work duties expected by men in order to prevent episodes of IPV are also likely to worsen leg swelling and disease outcomes.

Intimate partner violence had multiple negative impacts not only for these women living with podoconiosis, but also for their dependants. The most frequently reported effects of IPV on children were drop out from school, leaving the house at an early age, child work, child poverty and irrevocably damaged relationships between the children and the abusing partner. These impacts, in turn, help perpetuate the conditions of poverty that often determine the onset of the disease.

“*Now my son has grown and he asks him [husband] for money when he comes back home from Wellega. His father says ‘OK’, but delays for days. My son threatens to kill him unless he gives him money for his education. Now, the father visits his parents secretly, because he is afraid of his son. At this time, my son has stopped going to school because of lack of money or assistance*”

(Fikirte, aged 50)

“*He used to beat me as soon as he arrived home and because of this his parents decided to let me divorce from him but now I struggle to pay house rent…My daughter is now also a leg patient and cannot help so I beg for our house rent…*”

(Tsega, aged 45)

## Discussion

Intimate partner violence among women with podoconiosis takes diverse forms, including physical violence (enacted and threatened), verbal abuse, controlling and disempowering behaviors and sexual coercion. Particular examples include: beating with or without an object, threat of violence via use of weapons, insults, name calling, undermining, restricting choices, withholding money, extramarital affairs, forcing divorce, abandonment, obstruction of health care access and denial of equal rights and decision-making over shared resources. Global estimates published by the WHO indicate that about 1 in 3 (35%) women worldwide have experienced either physical or sexual intimate partner violence or non-partner sexual violence in their lifetime [6], and studies in Ethiopia indicate that between 50 and 71% of women experience one or more forms of spousal abuse at least once in their lifetime [19, 20, 30]. In the study region our research findings reveal that from the 105 women screened through HITS, 84 suffered from IPV (80%). IPV towards women living with podoconiosis is generally tolerated both by women and men, and more widely by the participants’ immediate social networks, including faith leaders (referred to by research participants as ‘the elders’). This is heavily influenced by women’s low recognition and status in the Ethiopian society in general, by a notable absence of formal IPV prevention and management services in the region and by a significant shortage of disability benefits or employment opportunities for women with disabilities.

All women living with podoconiosis experienced, to a greater or lesser extent, various and overlapping forms of IPV. Podoconiosis typically affects impoverished rural populations, which are also the populations most likely to experience intimate partner violence [30]. The social impact described here was consistent with studies that demonstrated IPV to be more common among certain demographic groups, including marginalized and disempowered women, women with lower incomes, women with disabilities [31, 32], and women who live in semi-urban or rural areas [6]. The abundant body of literature on HIV highlights that disease-associated stigma and discrimination are at the root of the IPV problem among women living with HIV [8, 10]. Our research however indicates that IPV among women living with podoconiosis is also heavily associated with these women’s decreased ability to fulfill the traditional gender roles in their respective households–coupled with men’s frustration at being married to an ‘unhealthy’ woman who struggles to fulfill their expectations of what ‘a woman should do’. Indeed, our findings are consistent with other regional studies focused on the intersection across IPV-disability-gender relations in that women living with this debilitating and disabling disease are expected to nevertheless fulfill the traditional gender roles in their households [32] and in that failure to complete household chores triggers episodes of IPV. Increasing local awareness of this debilitating disease, tackling the deeply rooted gendered roles and expectations at the household level and successfully challenging these women’s status quo in their households are urgent avenues for action.

Together with women’s reduced ability to work, poverty-related scarcities, the lack of formal IPV detection and management infrastructures and the general understanding of podoconiosis as a punishment from God or the result of magic forces, stigma is at the root of the complex relationship between podoconiosis and IPV. In endemic areas, podoconiosis is one of the commonest causes of stigmatization [12, 15]. The present study highlights the link between prejudice and discrimination against women living with podoconiosis, and shows that stigma is commonly enacted in the form of deprivation of emotional and material support [16, 17]. It also shows how intra-household discrimination and the associated deprivations faced by these women are a reflection of stigma against women (with or without debilitating diseases) in the broader society [32]. In order to truly limit disability and promote the wellbeing of women affected by podoconiosis proposed solutions therefore need to include societal level interventions regarding gender equality and women’s rights and entitlements.

Studies indicate that intimate partner violence is linked to several adverse health behaviors, chronic health conditions and mental health conditions, even after adjusting for demographic characteristics [33, 34]. Intimate partner violence among people living with podoconiosis is, therefore, also important because it influences disease outcome. The causal mechanism behind this lies in reduced access to resources (e.g. water and healthcare), but also on the effects of restricted decision-making of the affected women. Self-care is known to be a key determinant of outcomes in podoconiosis [35] and, as demonstrated, it is largely impeded by IPV. In turn, the negative impacts of IPV and podoconiosis on family members help perpetuate the conditions of poverty that often determine the onset of the disease. The development of accessible IPV prevention and management interventions is key for improving disease outcomes and for preventing disease onset in the region.

Many issues affect the relationship of IPV and podoconiosis, including the social acceptance of this issue in Ethiopia and the high alcohol consumption characteristic of the partners of the interviewed participants. As demonstrated, disease onset is not the main determinant of alcohol consumption but episodes of violence often occurred after partners had consumed alcohol. Our study also suggests that podoconiosis onset has influenced a transition from physical to psychological and financial violence. Podoconiosis has seemingly increased psychological violence and bullying despite the fact that physical violence commenced before most women suffered from podoconiosis. In fact, although IPV violence is tolerated amongst certain groups, physical violence against visibly diseased people is widely disapproved of, which could explain this transition. Tackling the high levels of alcohol consumption in the region is key for decreasing IPV, for improving disease outcomes and for preventing disease onset.

In this study, women’s experiences constituted “narratives of tolerance”. That is to say, women and members of their immediate social circles tolerated IPV, particularly in its early stages. When IPV became more severe, tolerance continued, even where there was cheating and abandonment. At these times women considered alternative pathways–for example, leaving home–but there were practical impediments to this. One reason for this was an overwhelming desire to prioritize the welfare of their children. A further driver was a downward socio-economic spiral where harvest produce, household income and social support decreased such that resources for change were minimal. Finally, there was evidence that belief in podoconiosis as divine punishment motivated tolerance. In addition to developing accessible IPV prevention and management interventions, there is an urgent need to tackle disease-associated taboo and stigma (e.g. through the development of educational programs focused on disease causation and disability management) and to develop sustainable strategies aimed at improving the socio-economic assets of women living with podoconiosis (e.g. housing and disability benefits, unemployment benefits, creation of livelihood opportunities for women with disabilities and of community-based support groups).

Other studies have shown that women tolerate IPV for the wellbeing of their children, and remain in the home for the sake of their children [36, 37] whilst hoping that the partner will change [30, 38]. Our research indicates that other reasons that prompt women to stay in violent relationships include fear of retaliation, lack of alternative means of economic support, lack of disability or unemployment benefits, lack of support from family and friends, stigma or fear of losing custody of children. Despite these barriers, many abused women eventually do leave their partners, often after multiple attempts and years of violence. In the WHO multi-country study, 19–51% of women who had ever been physically abused by their partner had left home for at least one night, and 8–21% had left two to five times [21, 30]. Factors associated with a woman trying to leave an abusive partner permanently appear to include an escalation in violence severity, a realization that her partner will not change, the existence of supportive networks (formal or informal) [39], access to formal or informal sources of economic support [40] and women’s recognition that the violence is affecting their children [36, 38]. However, in the study region, in face of seriously diminished supportive networks and the downward socio-economic spiral faced by women and their dependents after leaving the household, as shown, women often return to their abusive partners.

To our knowledge this is the first study to address the issue of IPV among women living with podoconiosis, but there is a dearth of information on the relationship between IPV and NTDs more generally. Given the aims of this study and its context-specificity, findings are not directly applicable to other contexts in which podoconiosis is prevalent, nor to other NTDs. More research is needed before useful generalizations can be promoted. More research is also needed to recognize the prevalence and burden of IPV among women living with podoconiosis compared to the general population, and to fully understand the complex intersections between IPV, disability, gender and poverty. Our study indeed highlights new avenues for action that can help limit disability and promote the wellbeing of people affected by podoconiosis in the study region (see above), but more research is needed for developing evidence-based tools to facilitate these changes in the study region. Further ways forward include the development of sustainable solutions to the problem of IPV amongst women living with long-term, disabling conditions in Ethiopia and beyond.

## Conclusion

Intimate partner violence is frequent among women affected by podoconiosis. The types of violence experienced by the women interviewed included psychological violence, physical violence, sexual punishment and diverse controlling behaviors. Women used different coping mechanisms and responded to intimate partner violence in different ways–heavily influenced by the impact of their circumstances on their mental wellbeing and self-esteem, the age of their children, their economic assets and by the support received from their inner circles. This study adds to the literature that women with podoconiosis are vulnerable to intimate partner violence and that IPV in turn worsens disease outcomes in many ways. However, income uncertainty and low employability due to the debilitating effects of podoconiosis often drive women to stay in abusive relationships. Tackling disease-associated taboo and stigma, developing accessible IPV interventions, working towards greater gender equality at the household and societal levels and developing sustainable strategies for improving the socio-economic assets of women affected by podoconiosis are all necessary to both prevent IPV and improve disease outcome.
